# Risk of Hemolytic Anemia in IBD Patients with Glucose-6-Phosphate Dehydrogenase Deficiency Treated with Mesalamine: Results of a Retrospective-Prospective and Ex Vivo Study

**DOI:** 10.3390/jcm12144797

**Published:** 2023-07-20

**Authors:** Maria Pina Dore, Giulia Tomassini, Chiara Rocchi, Milutin Bulajic, Monica Carta, Alessandra Errigo, Alberto Dimaggio, Federica Padedda, Giovanni Mario Pes

**Affiliations:** 1 Dipartimento di Medicina, Chirurgia e Farmacia, University of Sassari, Viale San Pietro 43, 07100 Sassari, Italy; giuliatomassini30@gmail.com (G.T.); a.errigo@studenti.uniss.it (A.E.); alberto.dimaggio@gmail.com (A.D.); federicapadedda92@gmail.com (F.P.); gmpes@uniss.it (G.M.P.); 2 Baylor College of Medicine, One Baylor Plaza Blvd., Houston, TX 77030, USA; 3 Gastroenterology and Endoscopy Unit, Mater Olbia Hospital, SS 125 Orientale Sarda, 07026 Olbia, Italy; chiara.rocchi@materolbia.com (C.R.); milutin.bulajic@materolbia.com (M.B.); 4 Department of Digestive Endoscopy, Fatebenefratelli Isola Tiberina-Gemelli Isola, Via di Ponte Quattro Capi 39, 00186 Roma, Italy; 5 Gastroenterology Unit, Santissima Annunziata Hospital, AOU-Sassari, Via San Nicola 6, 07100 Sassari, Italy; monica.carta@aouss.it; 6 Sardinia Longevity Blue Zone Observatory, 08040 Ogliastra, Italy

**Keywords:** sulfasalazine, mesalamine, 5-ASA, hemolytic anemia, glucose-6-phosphate dehydrogenase deficiency, inflammatory bowel disease

## Abstract

Background: Mesalamine is one of the most-used drugs in inflammatory bowel disease (IBD), especially ulcerative colitis. Regulatory agencies have listed mesalamine as an unsafe drug in subjects with glucose-6-phosphate dehydrogenase (G6PD) deficiency based on the risk of hemolysis, although scientific evidence is lacking. The occurrence of acute and/or chronic hemolytic anemia in IBD patients with G6PD deficiency exposed to mesalamine was evaluated. Methods: In this multicenter study, IBD patients with G6PD deficiency (cases) receiving mesalamine were retrospectively evaluated for acute, and prospectively for chronic, hemolysis. The presence of hemolytic anemia was based on red blood cell and reticulocyte count, hemoglobin, lactate dehydrogenase, unconjugated bilirubin, and haptoglobin levels. Cases were compared with controls (IBD patients with normal G6PD). Results: A total of 453 IBD patients (mean age 52.1 ± 16.0 years; 58.5% female) were enrolled. Ulcerative colitis was present in 75% of patients. G6PD deficiency was detected in 17% of patients. Oral mesalamine was used in 67.9% of ulcerative colitis and in 32.4% of Crohn’s disease cases. None of the 78 IBD patients with G6PD deficiency receiving mesalamine underwent hospitalization or specific treatment for acute hemolytic anemia. Relevant differences in chronic hemolysis markers were not observed in 30 cases compared with 112 controls receiving mesalamine (≤4500 mg/day). Marker modifications were also observed in mesalamine-free cases, consistent with the basal rate of erythrophagocytosis in G6PD deficiency. Ex vivo experiments showed the release of methemoglobin by G6PD deficient RBCs upon mesalamine challenge, only above 2.5 mg/mL, a concentration never reached in the clinical setting. Conclusions: This study provides, for the first time, evidence that mesalamine is safe in G6PD deficiency at a dosage of up to 4500 mg/day.

## 1. Introduction

Inflammatory bowel disease (IBD) is thought to result from the interaction of genetic and environmental factors that trigger an immunological response leading to intestinal inflammation. IBD includes ulcerative colitis and Crohn’s disease. The number of individuals diagnosed with IBD is steadily increasing worldwide. Drug therapy for IBD consists of 5-aminosalicylates (5-ASA or mesalamine), glucocorticoids, immunomodulators, biologic therapies, and antibiotics, as monotherapy, or in combination [1]. Unconjugated mesalamine is one of the most frequently used drugs to achieve remission, and for long-term maintenance, especially in ulcerative colitis. Mesalamine acts via several anti-inflammatory and immunosuppressive mechanisms [2,3,4,5]. Although oral formulations differ in the amount of active drug delivered to the colon [6], differences in efficacy and safety between compounds have not been reported [7]. Non-enteric coated or topical mesalamine also appears to be safe in pregnancy. Despite widespread use, mesalamine compounds have been reported to be unsafe in moderate to severe glucose-6-phosphate dehydrogenase (G6PD) deficiency by some Regulatory agencies and patient associations [8,9,10,11,12].

G6PD deficiency is one of the most common enzyme disorders, affecting 500 million individuals according to recent estimations, with great variation in prevalence across geographical areas [13]. The distribution of G6PD deficiency correlates with that of *Plasmodium falciparum*, such that regions in which malaria was, or is, endemic and areas with the highest prevalence of G6PD deficiency coincide. Sardinia has been reported to have the second highest prevalence of hereditary G6PD deficiency (4–35%) after Kurdish Jews (60–70%) [14,15].

Red blood cells (RBCs) of those with G6PD deficiency are very sensitive to oxidative injury, given the low amount of reduced nicotinamide adenine dinucleotide phosphate (NADPH) generated by the deficient enzyme. The disorder is X-linked and males inheriting a loss-of-function G6PD mutation result in hemizygosis for the defect and heterozygous females have approximately half of RBCs affected. Hundreds of G6PD variants have been described [16]. The severity of clinical manifestations depends on the specific mutation, and females with skewed X-inactivation may display a greater expression of the abnormal allele [17]. The G6PD Mediterranean variant (563C→T) is the most common variant in populations from the Mediterranean region, including Sardinia (>95%) [18] and the Middle East. Variants are categorized from class I to class V by the World Health Organization based on the magnitude of the enzyme deficiency [19]. The most dreaded clinical manifestations of G6PD deficiency are potentially life-threatening neonatal jaundice or acute hemolytic anemia, typically induced by medications, foods such as fava beans, infections, or chemical compounds, able to cause oxidative injury [20]. However, the majority of affected individuals may have only intermittent episodes of hemolysis or chronic hemolytic anemia. This study aimed to evaluate the occurrence of acute, and/or chronic hemolytic anemia in IBD patients with G6PD deficiency exposed to mesalamine.

## 2. Materials and Methods

### 2.1. Study Design

**Figure 1:**
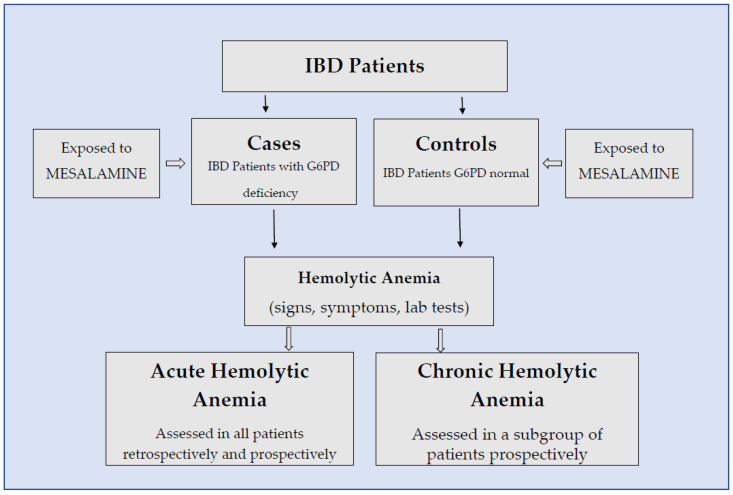
Study flow chart.

This was a retrospective-prospective multicenter and ex vivo study enrolling IBD patients with G6PD deficiency exposed to mesalamine. In order to assess the occurrence of acute, and/or chronic hemolysis, IBD patients with G6PD deficiency (cases) were compared with G6PD normal IBD patients (controls) (Figure 1).

### 2.2. Setting

Data were collected from IBD patients referred to the Gastroenterology section of (i) a teaching Hospital (University of Sassari, Sassari, Italy); (ii) a Civil Hospital (AOU-Sassari, Italy); and (iii) the Mater Hospital (Olbia, Italy). All patients were white from Northern Sardinia, a well-known and documented genetically homogeneous population [21]. 

### 2.3. Eligibility

Adult patients (aged ≥ 18 years) with a definite diagnosis of ulcerative colitis or Crohn’s disease, followed up for at least 6 months were enrolled. Exclusion criteria included: the presence of blood disorders―especially heterozygous beta-thalassemia that is frequent in Sardinia―malignancy, infectious diseases, and exposure to non-steroidal anti-inflammatory drugs (NSAIDs) or other compounds known to cause hemolysis at enrollment. Pregnant women were also excluded. The diagnosis of IBD was made according to the European and American Crohn’s and Colitis Organization guidelines, progressively developed and used in clinical practice. Informed consent was obtained from each participant. The study was approved by the Independent Ethics Committee, Comitato Etico Indipendente della AOU di Cagliari (Prot. PG/2021/17855), Italy.

### 2.4. Data Collection

Data from patients’ charts were recorded in a digital database. General information included sex, age, anthropometric parameters, cigarette smoking, and disease duration. Moreover, specific information was collected about IBD phenotype, anatomical localization, type of treatment and duration, hospitalizations, and surgeries for IBD or illness different from IBD, comorbidities, and medicines taken. Information about the specific type of mesalamine used, dosage, and schedule was also gathered. There was no Pharma participation in any phase of the study.

### 2.5. Hemolytic Anemia

Anemia was defined according to hemoglobin (Hgb) concentration or RBC count based on the following cutoffs: females Hgb < 11.9 g/dL; males Hgb < 14.0 g/dL. The presence of hemolytic anemia was assessed by laboratory tests such as increased reticulocyte count (normal range 0.5–2.5%; without recent bleeding, iron repletion, or administration of erythropoietin), high levels of lactate dehydrogenase (LDH) (range 125–220 U/L), unconjugated bilirubin (range 0.20–0.70 mg/dL), low haptoglobin levels (range 30–200 mg/dL), and the Coombs test if needed. Moreover, the serum iron (range 50–170 µg/dL), and serum ferritin (range 8–252 µg/mL) were measured. All tests were assessed in the same reference laboratory, at a given point during the study period, with the patient receiving mesalamine.

### 2.6. Determination of G6PD Activity

G6PD activity was determined by measuring the ratio between the catalytic activity of the enzyme and that of 6-phosphogluconate dehydrogenase (6PGD). This method improves the sensitivity of the biochemical tests, and identifies more accurately heterozygous females, with a reported sensitivity of 85.2% and a specificity of 97.4% [22]. The G6PD/6PGD ratio represents a normalized quantitative value to avoid interference of individual variations in the erythrocyte content of Hgb, the number of RBCs (for example, in microcytic and iron-deficient anemia), and the number of reticulocytes and leukocytes. Total G6PD deficiency was defined as G6PD/6PGD percent ratio < 10%, while the normal value was defined as G6PD/6PGD ≥ 85% and intermediate between 10% and 85% according to the manufacturer’s instruction (Nurex, Sassari, Italy) and based on the scientific literature [17,23].

### 2.7. Ex Vivo Chemotoxicity of Mesalamine

The effect of mesalamine was investigated ex vivo in G6PD deficient and normal RBCs. A 4-mL sample of peripheral venous blood was collected from anonymous male volunteers with G6PD normal or G6PD deficiency in tubes (Vacutainer^®^, Franklin Lakes, NJ, USA) containing K3EDTA as an anticoagulant and was used in all ex vivo experiments. A volume of 2 mL of blood sample was centrifuged at 1500× *g* to separate plasma and buffy coat from erythrocytes, and the latter were washed three times with sterile isotonic saline. An aliquot of the erythrocyte suspension was used to verify that in the G6PD deficient samples, the residual activity was less than 10%, as expected [18]. Based on preliminary experiments, the minimal concentration of mesalamine (Merck^®^, Readington, NJ, USA) capable of triggering hemolysis (based on methemoglobin formation in microtiter plates) was established. The drug was dissolved in isotonic saline in progressively increasing concentrations of 1.25, 2.5, 5, 10, 20, and 40 mg/mL, and 0.1 mL of G6PD normal and G6PD deficient RBC suspension was added to the drug solutions in duplicate. Total Hgb and methemoglobin levels in RBC were measured by the modified method of Dacie and Lewis [24]. The assay was an end-point reaction, and the mixture was incubated at 37 °C for 1 h until an absorbance plateau was reached. Results were expressed as percentages of methemoglobin.

### 2.8. Statistical Analysis

IBD patients were divided into those with Crohn’s disease, and those with ulcerative colitis. Significant variations of hemolytic anemia parameters between G6PD partial deficient and total deficient patients were not found in the preliminary analysis; therefore, individuals with severe and intermediate G6PD deficiency were grouped together as G6PD deficient. Treatment was coded by taking compounds such as (i) mesalamine, (ii) glucocorticoids, (iii) immunomodulators, (iv) biologic therapies, and (v) antibiotics. Use of mesalamine was stratified into (i) topic mesalamine, (ii) moderate amount (between 800 and 2500 mg/day ± topic mesalamine), and high amount of oral mesalamine (2500–4800 mg/day ± topical mesalamine). Patients underwent different and/or combined treatments over the course of their disease; therefore, only treatment with mesalamine alone or in combination was considered for the analysis. Additional subanalyses were performed according to the mesalamine formulation and treatment with oral budesonide and G6PD status. The number of hemolysis crises, requiring treatment or hospitalization during mesalamine exposure, was calculated according to the mesalamine dosage. The number of events was expressed per patient per year. Chronic hemolytic anemia was defined by the alteration of at least two lab tests among reticulocytosis, LDH, unconjugated bilirubin, and haptoglobin. The percent differences between G6PD deficient and normal subjects, in those exposed and not exposed to mesalamine were calculated. Age was grouped by decades. According to their smoking habits, patients were stratified as never, former, or current smokers. The body mass index (BMI) was calculated using the formula weight (kg)/height (m)^2^, and obesity was defined as a BMI ≥ 30 kg/m^2^. Continuous variables were expressed as mean ± standard deviation, and categorical variables as counts and percentages. The risk of hemolysis in patients with or without G6PD deficiency exposed to mesalamine was estimated by using a Pearson chi-square test with the procedure available in the SPSS Statistical Package (version 22.0, Chicago, IL, USA). The sample size was calculated using the formula N_0_ = *Z*^2^*p*(1 − *p*)/*d*^2^ where N_0_ is the sample size, *Z* is the *Z*-score equal to 1.96, *d* is precision equal to 0.05, and *p* is the expected prevalence, obtaining the value of 138. Results were considered significant when two-tailed *p*-values were <0.05.

## 3. Results

A total of 457 IBD patients (mean age of 52.1 ± 16.4 years; 58.5% female) were enrolled. Four patients were excluded from the analysis, two patients for the presence of autoimmune anemia (both had ulcerative colitis), and two were due to Gilbert syndrome. Among IBD patients, ulcerative colitis was present in 75% and Crohn’s disease in 25% (Table 1).

The fifth decade comprised the highest prevalence of IBD patients for both phenotypes. Overweight and obesity were more frequent in ulcerative colitis, whereas a BMI lower than 25 kg/m^2^ was more prevalent in Crohn’s disease. Never smokers or former smokers were the majority compared with current smokers (82.3% and 17.7%). The most commonly prescribed treatment was oral mesalamine (67.9% and 32.4%) in ulcerative colitis and Crohn’s disease, respectively, including a minority of patients receiving sulfasalazine (three cases). Disease duration in months and IBD localization are shown in Table 1.

In the retrospective part of the study, the prevalence of G6PD deficiency was 12.4%, similar to the frequency reported in the general Sardinian population [18]. Instead, in the subgroup of IBD patients recruited prospectively, G6PD deficiency was observed in 14.3% and 27% of patients with ulcerative colitis and Crohn’s disease, respectively. The frequency of G6PD deficiency was relatively higher probably because patients with G6PD deficiency were more prone to participate in the study.

In Table S1 of the Supplementary Materials, the demographic and clinical features of the IBD cohort studied according to G6PD status are shown.

Overall, mesalamine was well tolerated. One young female patient developed a mild alteration of the liver function tests and two male patients a mild pancreatitis. In all three cases, they recovered by reducing the mesalamine dosage from 4800 mg to 2400 mg. Side effects related to the treatment according to G6PD status are reported in Table S2. Dyspepsia, nausea, and abdominal pain were the most common complaints, in all cases mild and without significant differences between normal and G6PD deficient patients (8.9% vs 13.6%).

Not one IBD patient with G6PD deficiency treated with mesalamine underwent hospitalization or specific treatment for acute hemolytic anemia. 

Table 2 lists laboratory parameters from 164 IBD patients prospectively enrolled according to the G6PD status based on mesalamine exposure. All parameters resulted slightly modified in patients with G6PD deficiency compared with G6PD normal patients, with the only exception of unconjugated bilirubin levels that were significantly higher in those receiving high dosage mesalamine and with G6PD deficiency compared with G6PD normal patients. Interestingly, similar changes in laboratory parameters, suggestive of a basal low-grade chronic hemolytic anemia, were also observed in IBD patients with G6PD deficiency unexposed to mesalamine (Table 2).

In this subgroup, the only statistically significant modification was observed for the Hgb levels in patients not taking mesalamine (14.7 ± 1.0 vs. 12.6 ± 0.9 g/dL; *p* < 0.001).

In order to evaluate chronic hemolysis related to the type of mesalamine formulation in patients with G6PD deficiency, an additional subanalysis was performed according to the mesalamine formulation and oral budesonide (Table S3).

Although some chronic hemolytic markers were found significantly modified for certain mesalamine formulations and oral budesonide compared with other formulations, the small number of IBD patients G6PD normal and deficient did not allow definitive conclusions or interpretations of the results. Similarly, the excessive dilution of the numbers did not allow further sub-analyses relating to the specific mesalamine formulation according to the dosage or in combination with other ongoing treatments such as statin, proton pump inhibitors, antihypertensive, etc. 

**Figure 2:**
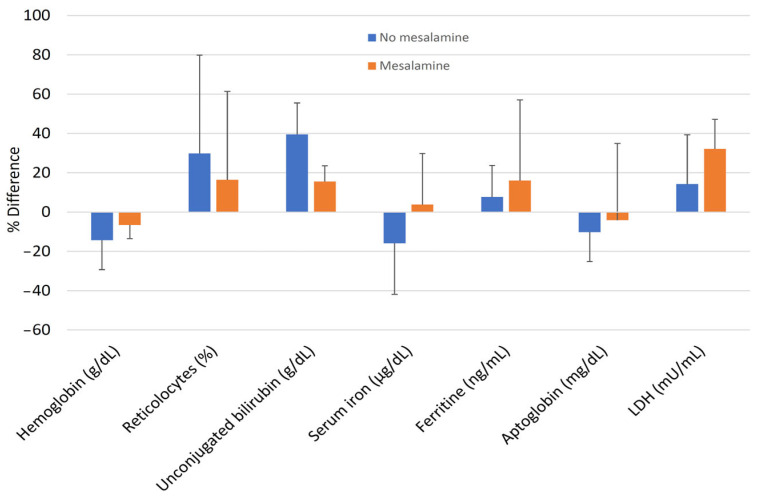
Differences (deltas) of hemolysis markers between IBD patients with G6PD-deficiency according to mesalamine therapy.

The percent differences (deltas) of hemolysis markers between mesalamine-free IBD patients and those receiving mesalamine at high dosages were similar and not significantly different from zero (Figure 2).

### Ex Vivo Chemotoxicity of Mesalamine

**Figure 3:**
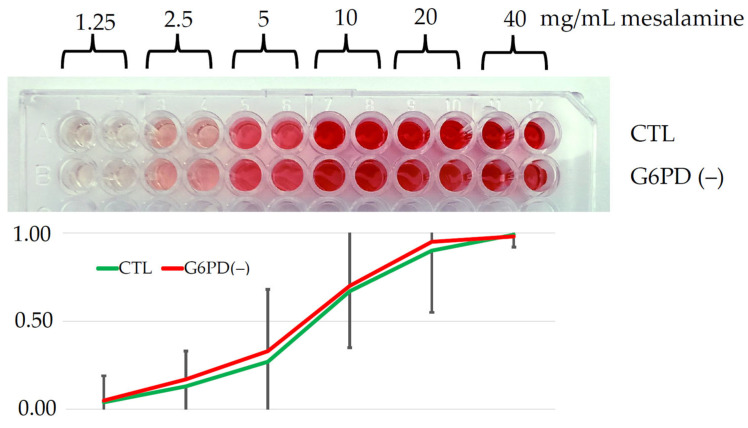
The microtiter plate shows the ex vivo chemotoxicity of mesalamine in patients with and without G6PD deficiency. The two well lines represent glucose-6-phosphate dehydrogenase G6PD(−) deficient erythrocytes, the second well line contains G6PD normal erythrocytes. The curves indicate the absorbance related to the release of methemoglobin upon mesalamine challenge in both groups (see explanation in the text).

Methemoglobin production (a hemolysis index) was similar between G6PD deficient and G6PD normal erythrocytes upon challenge with increasing concentrations of mesalamine. After 1 h of incubation, no appreciable difference in absorbance between G6PD deficient and control samples was observed (*p* > 0.05) (Figure 3). Significant damage to erythrocytes and hemolysis occurred above the concentration of 2500 μg/mL in both G6PD deficient samples and controls. Usually, in a clinical setting, circulating mesalamine reaches levels lower than 1000 ng/mL [25], (i.e., 10^3^ times lower than the minimal concentration capable of causing hemolysis in vitro).

## 4. Discussion

Mesalamine and sulfasalazine are frequently used in clinical practice, especially to treat ulcerative colitis, diverticular disease, and mild Crohn’s disease confined to the colon [26]. In the past, sulfasalazine was used to treat rheumatoid arthritis, but nowadays it is recommended only for treating Crohn’s disease with joint involvement. Unconjugated mesalamine is usually preferred because sulfapyridines are responsible for the majority of side effects, although an increase in mesalamine intolerance has been reported [27]. The clinical efficacy of mesalamine is not yet completely understood. Inhibition of proinflammatory cytokine [2], prostaglandin, and leukotriene synthesis [3]; inhibition of polymorphonuclear leukocytes function and adhesion [4]; and an overall immunosuppressive role [5] are a few of the suggested mechanisms of action of mesalamine. Moreover, mesalamine is recognized as a potent antioxidant and free radical scavenger of the reactive metabolites of oxygen produced in the intestinal mucosa [28]. Mesalamine is also considered as chemotherapy in the prevention of colorectal cancer, particularly in those patients with genetic predisposition, and familial polyposis [29]. However, the “salicylic” moiety of mesalamine, similar to the acetylsalicylic acid, has long been considered responsible for hemolytic events in G6PD deficient patients, although the two molecules differ greatly. In fact, acetylsalicylic acid is an acetyl donor able to acetylate some lysine residues of G6PD protein located in a critical position for catalytic activity [30]. Mesalamine has a much lower ability to transfer the acetyl group because it is linked to a nitrogen atom [31].

Our retrospective, prospective, and ex vivo study demonstrated that any type of mesalamine formulation, even at the high dosages (e.g., 4.5 g), recommended to induce remission in patients with ulcerative colitis, displays a safe profile also in subjects with G6PD deficiency Mediterranean variant. No hemolytic crises were observed in our patients, although mild changes in hemolysis markers were detected in otherwise asymptomatic patients with enzymopathy. Moreover, the alterations in hemolysis markers were observed among IBD patients with G6PD deficiency unexposed to mesalamine, suggesting the existence of a basal low-grade chronic hemolysis. Interestingly, in the mesalamine-free IBD patients, a significant decrease in Hgb levels was observed. It is likely that in those IBD patients requiring more intensive treatment, such as immunomodulators or biologics, the clinical severity may have caused mucosal bleeding. In addition, the use of unsafe over-the-counter drugs (e.g., NSAIDs) on demand, or exposure to some harmful chemicals cannot be excluded. Moreover, a background of hemocatheretic activity in G6PD deficient subjects, given the instability of the erythrocyte membrane, intrinsically present in carriers of the enzymatic defect, has been well documented (extravascular hemolysis) [16,17].

The results of our study were predictable considering that mesalamine has been used for decades by specialists and physicians, despite being listed as contraindicated by some regulatory agencies, and patient associations [8,9,10,12]. In fact, agreement on safe or unsafe drugs in G6PD-deficiency is not universal as different sources provide different lists extrapolated by the literature, without rigorous proof. This seems to be the case with mesalamine. For example, there is only one case report published in the second half of the 1900s reporting hemolytic events related to the assumption of sulfasalazine in patients affected by G6PD deficiency [32]. In 2023, Gammal et al. [33] published the implemented guideline for medication use in the context of G6PD deficiency genotype based on published literature, and according to the United States Food and Drug Administration (FDA), European Medicines Agency (EMA), Pharmaceuticals and Medical Devices Agency (PMDA)–Japan, extrapolated from the www.pharmgkb.org. The authors reported a risk level for sulfasalazine in G6PD deficient subjects “low-to-no” with a strength of recommendation “optional” meaning: “the desirable effects are closely balanced with undesirable effects, or the evidence is weak or based on extrapolations…”, and for mesalamine: no recommendation “There is insufficient evidence, confidence, or agreement to provide a recommendation to guide clinical practice at this time” [33]. The findings of our study corroborate the conclusion of the aforementioned authors.

The present study has some limitations. First, the retrospective part of the design may include some biases, although missing data about major hemolytic events in IBD patients with G6PD deficiency seems unlikely as patients were followed in our gastroenterology section with a median of at least 4–6 visits per year. Second, DNA analysis to exclude skewed inactivation of the X chromosome in heterozygous females was not available, although, given the results, the additional analysis should not dramatically change the overall findings. An additional limitation may be the small number of prospectively recruited patients, which did not allow further analyses such as, for example, possible differences between males and females, and between ulcerative colitis and Crohn’s, among others. In any case, this pilot study may pave the way for studies with larger cohorts.

## 5. Conclusions

In conclusion, this study provides, for the first time, compelling evidence that mesalamine, in any formulation, is safe in G6PD deficiency also at a dosage of up to 4800 mg/day. These results may prompt additional prospective research in larger cohorts.

## Figures and Tables

**Table 1:** Descriptive statistics and clinical features of 453 study participants with inflammatory bowel disease (IBD).

Variables	Ulcerative Colitis (*n* = 342)	Crohn’s Disease (*n* = 111)
Sex, *n* (%)		
Female	191 (55.8)	70 (63.1)
Male	151 (44.2)	41 (36.9)
Age (years)	51.9 ± 16.6	52.5 ± 15.4
Age distribution, *n* (%)		
<30	39 (11.4)	9 (8.1)
30–39	52 (15.2)	16 (14.4)
40–49	56 (16.4)	18 (16.2)
50–59	75 (21.9)	31 (27.9)
60–69	64 (18.7)	24 (21.6)
70–79	45 (13.2)	9 (8.1)
≥80	11 (3.2)	4 (3.6)
Body mass index, *n* (%)		
<25 kg/m^2^	191 (55.9)	75 (67.6)
25–29 kg/m^2^	113 (33.0)	27 (24.3)
≥30 kg/m^2^	38 (11.1)	9 (8.1)
Cigarette smoke, *n* (%)		
No	171 (50.0)	53 (47 7)
Former smoker	126 (36.8)	23 (20.7)
Current smoker	45 (13.2)	35 (31.5)
Treatment, *n* (%)		
Topical	31 (9.1)	8 (7.2)
Mesalamine per os	232 (67.8)	36 (32.4)
Immunomodulators	51 (14.9)	30 (27.0)
Biologic	28 (8.2)	37 (33.3)
Disease duration, months (%)		
6–9	90 (27.6)	20 (19.4)
10–19	206 (63.2)	73 (70.9)
20–29	26 (8.0)	7 (6.8)
≥30	4 (1.2)	3 (2.9)
IBD localization (%)		
Distal proctitis	12 (3.5)	0
Ulcerative left colitis	187 (54.7)	0
Pancolitis	143 (41.8)	0
Ileocolic Crohn’s	0	80 (72.1)
Colic Crohn’s	0	16 (14.4)
Crohn’s with fistulae	0	15 (13.5)
G6PD ^1^ deficiency, *n* (%)		
No	294 (85.7)	81 (72.9)
Yes	48 (14.3)	30 (27.0)

^1^ Glucose-6-phosphate dehydrogenase.

**Table 2:** Markers of hemolitic anemia among 164 IBD patients according to mesalamine dosage, and G6PD status.

Variables (Normal Range)	No Mesalamine		Topic Mesalamine		Moderate Amount ^#^ of Mesalamine		High Amounts ^§^ of Mesalamine	
	G6PD Normal (*n* = 10)	G6PD Deficiency (*n* = 12)	G6PD Normal (*n* = 10)	G6PD Deficiency (*n* = 10)	G6PD Normal (*n* = 78)	G6PD Deficiency (*n* = 12)	G6PD Normal (*n* = 24)	G6PD Deficiency (*n* = 8)
Hemoglobin ^†^ (g/dL)	14.7 ± 1.0	12.6 ± 0.9 **	13.7 ± 0.7	12.9 ± 0.8	13.9 ± 1.5	13.7 ± 0.9	13.7 ± 1.4	12.8 ± 0.7
Reticulocytes (0.5–2.5%)	1.34 ± 0.49	1.74 ± 0.84	1.4 ± 0.5	1.81 ± 0.57	1.26 ± 0.43	1.44 ± 0.42	1.34 ± 0.42	1.56 ± 0.47
Unconjugated bilirubin (0.20–0.70 g/dL)	0.43 ± 0.16	0.60 ± 0.43	0.54 ± 0.20	0.52 ± 0.31	0.35 ± 0.06	0.46 ± 0.37	0.45 ± 0.18	0.52 ± 0.18 *
Serum iron (50–170 µg/dL)	86.0 ± 26.0	72.3 ± 24.0	84.2 ± 20.1	89.9 ± 0.27	80.4 ± 33.0	75.7 ± 17.6	86.4 ± 29.2	89.7 ± 22.9
Ferritine (8–252 µg/mL)	94.9 ± 61.8	102.2 ± 36.2	82.0 ± 75.8	111.2 ± 36.2	100.7 ± 73.2	109.6 ± 35.2	77.0 ± 84.0	89.4 ± 27.8
Haptoglobin (30–200 mg/dL)	136.8 ± 69.8	122.8 ± 27.1	164.3 ± 63.4	121.6 ± 27.1	173.2 ± 74.6	255.3 ± 74.2	136.6 ± 62.3	131.0 ± 26.5
Lactate dehydrogenase (125–220 U/L)	163.3 ± 25.0	186.6 ± 23.9	181.6 ± 24.7	198.2 ± 29.4	198.3 ± 94.6	211.5 ± 92.1	141.2 ± 22.0	166.7± 27.9

**^#^** Moderate amount (between 800 and 2500 mg/day ± topic mesalamine); **^§^** High amount of oral mesalamine (2500–4800 mg/day ± topical mesalamine). ^†^ Hemoglobin ranges: females <11.9 g/dL; males <14.0 g/dL. * *p* < 0.05, ** *p* < 0.001

## Data Availability

Data will be available to the corresponding author upon reasonable request.

## Supplementary Materials

The following supporting information can be downloaded at: https://www.mdpi.com/article/10.3390/jcm12144797/s1, Table S1: Demographics and clinical features of 453 study participants with inflammatory bowel disease (IBD) stratified according to Glucose-6-phosphate dehydrogenase status; Table S2: Side effects complained by IBD patients treated with oral mesalamine as monotherapy according to the G6PD Status; Table S3: Markers of hemolytic anemia among 158 IBD patients according to oral mesalamine formulation, oral budesonide and G6PD status.
