# Supplementary material for: Risk of Hemolytic Anemia in IBD Patients with Glucose-6-Phosphate Dehydrogenase Deficiency Treated with Mesalamine: Results of a Retrospective-Prospective and Ex Vivo Study
Source: J Clin Med. 2023 Jul 20;12(14):4797. doi: 10.3390/jcm12144797 (PMC10381559; doi:10.3390/jcm12144797)
Supplement: Supplementary file 1 [file jcm-12-04797-s001.zip › jcm-2474802-SI.pdf]

Table S1. Demographics and clinical features of 453 study participants with inflammatory bowel disease (IBD) stratified according to Glucose-6-phosphate dehydrogenase status.

**Variables**	**G6PD [1] normal** (n=375)	**G6PD [1] deficiency** (n=78)
Sex, n (%)		
Female	208 (55.5)	52 (66.7)
Male	167 (44.5)	26 (33.3)
Age (mean, years)	52.4 ± 16.4	50.9 ± 16.6
Age distribution, n (%)		
< 30	39 (10.4)	9 (11.5)
30 - 39	53 (14.1)	14 (17.9)
40 - 49	62 (16.5)	11 (14.1)
50 - 59	88 (23.5)	18 (23.1)
60 - 69	75 (20.0)	14 (17.9)
70 - 79	45 (12.0)	10 (12.8)
≥ 80	13 (3.5)	2 (2.6)
Body mass index, n (%)		
< 25 kg/m²	258 (68.8)	45 (57.7)
25 – 29 kg/m²	92 (24.5)	25 (32.1)
≥ 30 kg/m²	25 (6.7)	8 (10.3)
Cigarette smoke, n (%)		
No	233 (56.8)	53 (67.9)
Former smoker	121 (32.3)	19 (24.4)
Current smoker	41 (10.9)	6 (7.7)
Treatment#, n (%)		
Topical	26 (6.9)	13 (16.6)
Mesalamine per os	247 (65.8)	47 (60.3)
Immunomodulators	61 (16.2)	20 (25.6)
Biologic	54 (14.4)	11 (14.1)
Disease duration, months (%)		
6 – 9	95 (25.3)	22 (28.2)
10 – 19	238 (63.5)	47 (60.3)
20 – 29	34 (9.1)	5 (6.4)
≥ 30	8 (2.1)	4 (5.1)
IBD type, n (%)		
Crohn's	81 (21.6)	30 (38.5)
Ulcerative colitis	294 (78.4)	48 (61.5)
IBD localization (%)		
Distal proctitis	11 (2.9)	1 (1.3)
Ulcerative left colitis	150 (40.0)	37 (47.4)
Pancolitis	123 (32.8)	20 (25.6)
Ileocolic Crohn's	61 (16.3)	19 (24.4)
Colic Crohn's	16 (4.3)	0 (0.0)
Crohn's with fistulae	14 (3.7)	1 (1.3)

[1] Glucose-6-phosphate dehydrogenase; [#] Drugs were used in different combinations

Table S2. Side effects complained by patients with inflammatory bowel disease treated with oral mesalamine as monotherapy according to the G6PD Status

Side effects	G6PD [1] Normal (n=101)	G6PD Deficiency (n=22)
Dispepsia	3	1
Nausea	2	1
Abdominal pain	2	1
Abdominal disconfort	1	0
Abdominal bloating	1	0
Headache	1	0
Constipation	1	0
Pruritus	1	0

[1] Glucose-6-phosphate dehydrogenase

Table S3. Markers of hemolytic anemia among 158 patients with inflammatory bowel disease according to oral mesalamine formulation, oral budesonide and glucose-6-phosphate dehydrogenase status.

**Variables (normal range)**	**Multimatrix**		**Delayed release enteric-coated tablet pH dependent**		**Controlled release capsule Time reales**		**Oral Budesonide**	
	**G6PD normal** (n=46)	**G6PD deficiency** (n=10)	**G6PD normal** (n=31)	**G6PD deficiency** (n=7)	**G6PD normal** (n=25)	**G6PD deficiency** (n=3)	**G6PD normal** (n=7)	**G6PD deficiency** (n=4)
Hemoglobin† (g/dL)	14.0 ± 1.2	13.2 ± 1.1	14.3 ± 1.6	12.2 ± 0.8 *	13.6 ± 0.8	13.5 ± 0.4	14.1 ± 0.9	12.3 ± 1.0**
Reticolocytes (0.5-2.5%)	1.21 ± 0.34	1.67 ± 0.75 *	1.29 ± 0.46	2.00 ± 1.13	1.42 ± 0.71	1.10 ± 0.14	1.29 ± 0.59	1.76 ± 0.94
Unconjugated bilirubin (0.20-0.70 g/dL)	0.50 ± 0.49	0.53 ± 0.22	0.46 ± 0.22	0.37 ± 0.04	0.44 ± 0.10	0.94 ± 0.65 *	0.52 ± 0.21	0.63 ± 0.45
Serum iron (50-170 μg/dL)	73.7 ± 27.5	77.4 ± 22.4	89.6 ± 35.9	75.3 ± 18.9	97.3 ± 30.2	55.0 ± 14.1	86.1 ± 28.0	72.9 ± 23.2
Ferritine (8-252 μg/mL)	101.2 ± 54.9	101.3 ± 38.8	106.4 ± 85.5	112.7 ± 23.7	122.7 ± 134.4	100.1 ± 94.5	93.3 ± 62.1	100.1 ± 38.1
Haptoglobin (30-200 mg/dL)	171.4 ± 80.6	197.8 ± 92.7	178.8 ± 55.7	147.3 ± 22.5	130.3 ± 54.2	95.0 ± 7.0	139.2 ± 67.9	120.8 ± 28.1
Lactate dehydrogenase (125-220 U/L)	200.6 ± 51.3	193.2 ± 29.6	123.7 ± 31.3	173.2 ± 37.8 *	179.0 ± 1.41	213.6 ± 136.1	168.3 ± 24.1	187.5 ± 24.1

* $p<0.05$; ** $p<0.001$
